# Identification of a Short Region on Chromosome 6 Affecting Direct Calving Ease in Piedmontese Cattle Breed

**DOI:** 10.1371/journal.pone.0050137

**Published:** 2012-12-04

**Authors:** Silvia Bongiorni, Giordano Mancini, Giovanni Chillemi, Lorraine Pariset, Alessio Valentini

**Affiliations:** 1 Department for Innovation in Biological, Agro-food and Forest Systems, University of Tuscia, Viterbo, Italy; 2 CASPUR, Inter-University Consortium for the Application of Super-Computing for Universities and Research, Rome, Italy; Morehouse School of Medicine, United States of America

## Abstract

Calving in cattle is affected by calf morphology and by dam characteristics. It is described by two different traits: maternal calving ease, which is the ability to generate dams with good physiological predisposition to calving, and direct calving ease, which is the ability to generate calves that are easily born. The aim of this study was to identify regions of cattle genome harboring genes possibly affecting direct calving ease in the Piedmontese cattle breed. A population of 323 bulls scored for direct calving ease (EBV) was analyzed by a medium-density SNP marker panel (54,001 SNPs) to perform a genome-wide scan. The strongest signal was detected on chromosome 6 between 37.8 and 38.7 Mb where 13 SNPs associated to direct calving ease were found. Three genes are located in this region: *LAP3*, encoding for a leucine aminopeptidase involved in the oxytocin hydrolysis; *NCAPG*, encoding for a non-SMC condensin I complex, which has been associated in cattle with fetal growth and carcass size; and *LCORL*, which has been associated to height in humans and cattle. To further confirm the results of the genome-wide scan we genotyped additional SNPs within these genes and analyzed their association with direct calving ease. The results of this additional analysis fully confirmed the findings of the GWAS and particularly indicated *LAP3* as the most probable gene involved. Linkage Disequilibrium (LD) analysis showed high correlation between SNPs located within *LAP3* and *LCORL* indicating a possible selection signature due either to increased fitness or breeders’ selection for the trait.

## Introduction

Reproductive health in cattle is an economically important trait and calving is a key element to improve productivity and animal welfare. Calving difficulty, also known as dystocia, has negative effects on the sustainability and profitability of a herd by adding considerable costs for veterinary care, hampering the next conception, and worsening the animal welfare. Calving is influenced by many environmental and genetic factors: in cattle it is affected by calf morphology [1] and by dam characteristics [1] and can be described by two traits: maternal calving ease and direct calving ease. Both refer to calving without human intervention, the first attributed to the mother and the second to the calf. In addition to birth weight, other important factors as calf shape, the size of cow pelvic area or the gestation length may contribute to calving ease [2]. One of the major causes of dystocia is a disproportion between size of the calf at birth and the cows’ birth canal. Thus, the cow physiological predisposition to birth and the calf morphology are the key genetic factors which can be improved by selection [3]. The Estimated Breeding Values (EBVs) for calving ease used in breeding plans are measured from three sets of records: calving ease score, birth weight and gestation length. Several studies have identified QTLs influencing calving traits [4], [5], [6], [7], [8], [9]. The estimation of EBV and the identification of genes significantly associated with calving performance is a challenging task since the traits show low heritability and the recording is made on scales which are subject to breeder’s interpretation. The recent availability of cattle genome-wide SNP panels could potentially overcome these drawbacks [10]. The use of large-scale single nucleotide polymorphism (SNP) genotyping and genome-wide association (GWA) studies allow to identify genomic regions and hopefully mutations that underlie the desired performance.

The aim of this study was to identify regions of the bovine genome possibly affecting calving performance. We used most of the records registered in the Piedmontese breed, which is known to be at risk of dystocia like all breeds that carry harmful mutations in the myostatin gene [11]. In our work, a medium density SNP marker panel (54,001 SNPs) was used to investigate the genome of 323 Piedmontese bulls for identifying chromosomal regions associated with calving traits. This genome-wide scan let us to discover a 0.9 millions of base pairs (Mbp) region of chromosome 6 (37.8–38.7 Mbp, on the Btau_4.0), associated with direct calving ease and to pinpoint with high probability genes associated to the trait.

## Results

### Data Editing

**Figure 1:**
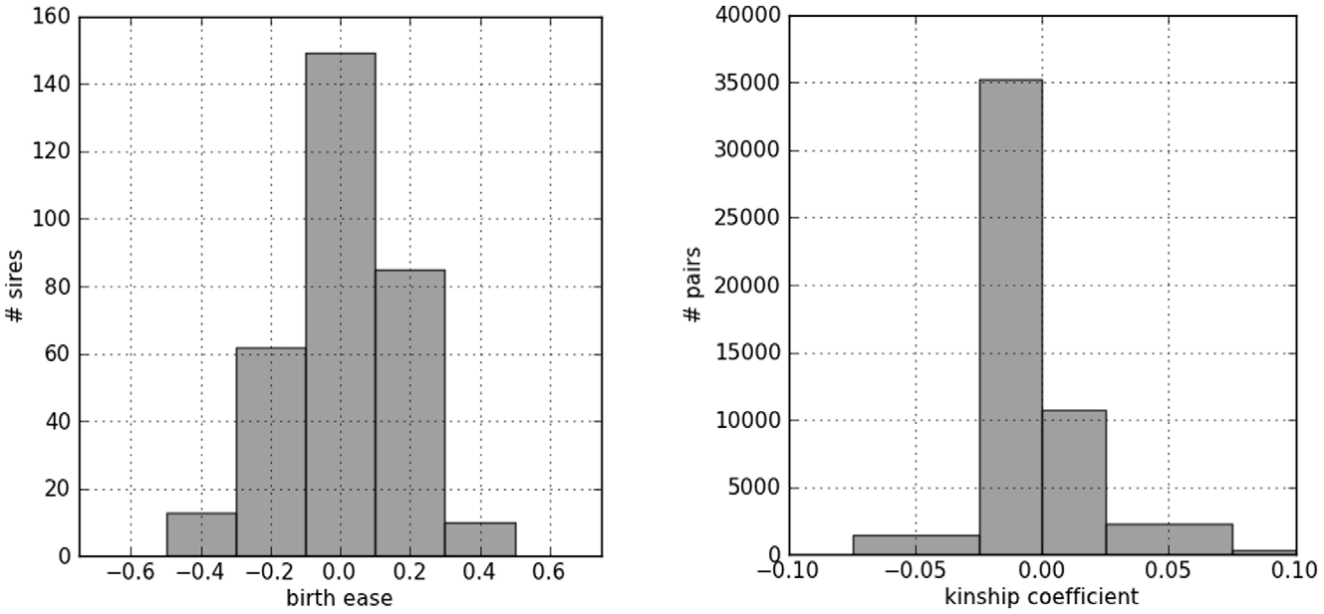
Left Panel: histograms of EBVs for direct calving ease distributed in five equally spaced classes. Right panel: histogram of kinship coefficients between sires in five equally spaced classes. In both panels, the number of sires belonging to each class is plotted as a function of classes.

Three of the 323 sires were excluded for having a genotype call rate lower than 95% and two for having a too high autosomal heterozygosity. As for markers, 506 out of the 54,001 SNPs of the chip were excluded because the ratio of missing genotypes was higher than 5%; 10870 because showed minor allele frequency below 2%; 1372 because they were out of Hardy-Weinberg Equilibrium (HWE, P<0.01). The final dataset was thus formed by 318 sires and 41333 SNPs. Figure 1 (left panel) shows the distribution of EBV values for direct calving ease partitioned in five equally spaced classes; an acceptable approximation of a normal distribution can be observed for direct calving ease, with the three central intervals accounting for 75% of sample size; a tiny extreme negative tail which is not mirrored by positive values can be observed.

### Genome-wide Scan

**Figure 2:**
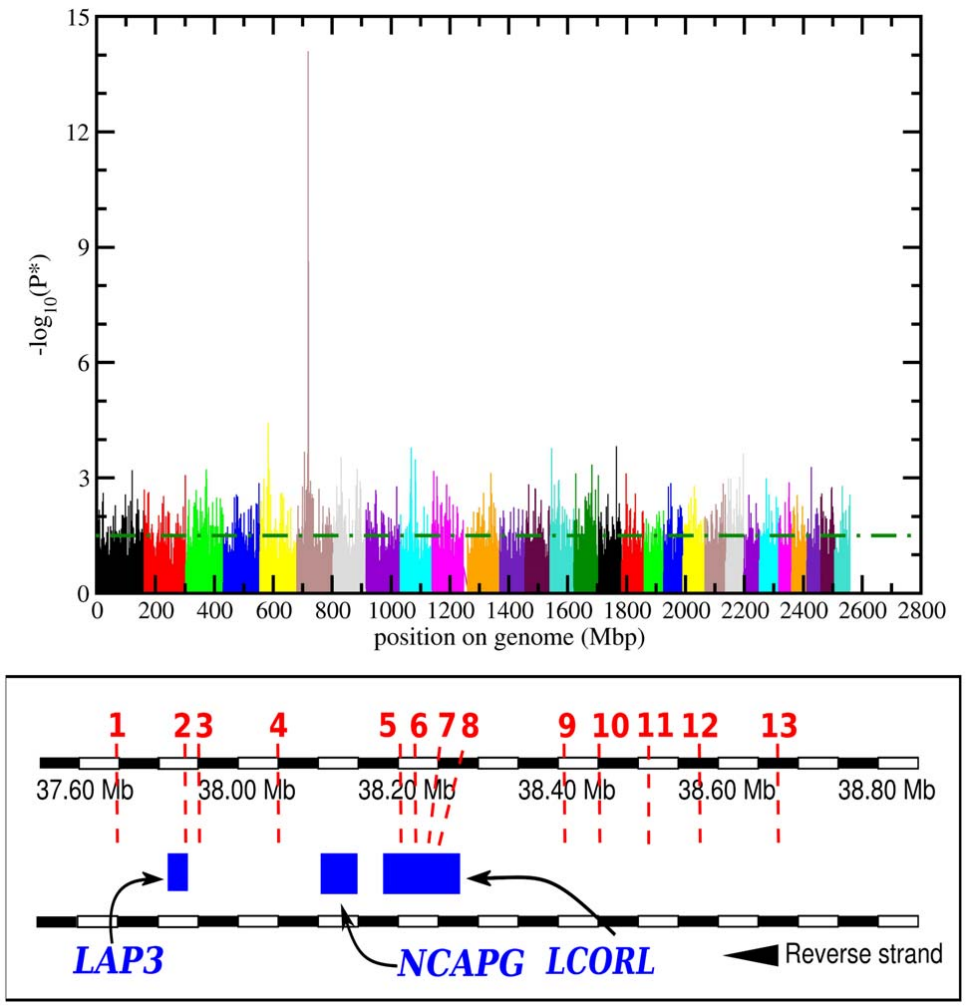
Upper panel: log significance levels of SNPs association with direct calving ease for the entire filtered dataset before the correction for inflation. Each autosome is represented with a different colour and position is calculated along the entire genome length. Values above the dot-dashed green line denote a P*<0.05. Lower panel: The chromosomal region spanned by the 13 markers listed in Table 1. The approximate position and size of genes located in the region (blue rectangles) together with the position of each SNP (red numbers and red dashed lines) are shown.

Distribution of genomic kinship values is shown in Figure 1 (right panel); most sires share a genomic kinship value in the range ±0.025 and higher values are very uncommon in the sample; thus the chosen statistical approach coupled with genomic control should be able to correctly take into account the population structure while conserving enough statistical power. Results of the genome-wide scan for direct calving ease traits are summarized in the Manhattan plot of Figure 2 (upper panel). Among the 41333 polymorphisms of the Illumina BovineSNP50 retained after quality control, 2030 (roughly 0.5%) were associated to direct calving ease. P-values ranged from 0.05 (chosen as the maximum value to define a SNP as significantly associated to direct calving ease) and 8.17E-15 (see Table 1). A very sharp peak containing several SNPs with very high -log_10_(P*) values can be observed on the first half of BTA (*Bos taurus* autosome) 6, between 37.8 and 38.7 millions of base pairs (Mbp) (i.e. between 715 and 720 Mbp relatively to total genome length, Btau_4.0). The inflation factor λ obtained for the test was 1.03 showing that, given the number of markers, residual inflation was acceptable; a qq-plot of P-values prior to λ normalization is shown in Figure S1 in the Supplementary Materials. Correction for λ yielded a total of 1876 significant SNPs; of these, 1833 had a λ-corrected P-value in the interval 0.05>P≥0.001 and 43 had a λ-corrected P-value <0.001. Significance levels (unadjusted: P*, and adjusted with the Benjamini-Hochberg method, Q*, see Materials and Methods section) of these SNPs, SNP names and their position on BTA6 according to Illumina BovineSNP50 specifications, and the gene in which they are located within (if any) are shown in Table 1. A clear association of the identified chromosomal region with the target trait can be observed, as statistically confirmed by several markers showing a P-value <10^−7^ also after the correction for multiple testing. Allelic effects of the minor allele (last column of Table 1) are small and positive except for two markers (Hapmap26308-BTC-057761 and ARS-BFGL-NGS-45457). Figure 2 (lower panel) shows the region on BTA6 spanned by the 13 most significant SNPs showed in the Manhattan plot (Figure 2 upper panel) and listed in Table 1; one marker (Hapmap26308-BTC-057761) is within the sequence of the *LAP3* gene while four (Hapmap 27083-BTC-041166, 23507-BTC-041133, 31285-BTC-041097, 33628-BTC-041023) are located in the *LCORL* gene. Average Linkage Disequilibrium (LD) estimation for all markers in the selected region yielded r^2^ = 0.426 but higher correlation was observed between SNPs within the *LCORL* gene (r^2^ = 0.637) and between SNPs located in *LCORL* and *LAP3* (r^2^ = 0.571).

**Table 1:** Single nucleotide polymorphisms associated to direct calving ease and located on BTA6 in the region comprised between 37 and 39 millions of base pairs sorted according to their position on the Illumina BovineSNP50 panel.

SNP name	Position (bp)	gene	P*	Q*	fB	βB
Hapmap30134-BTC-034283	37,852,401	NA	2.38E-09	1.94E-05	0.45	8.43E-02
Hapmap26308-BTC-057761	37,963,148	*LAP3*	8.17E-15	3.38E-10	0.40	−1.19E-01
ARS-BFGL-NGS-112812	38,014,255	NA	3.87E-06	1.60E-02	0.35	6.85E-02
ARS-BFGL-NGS-45457	38,102,328	NA	7.97E-13	1.65E-08	0.45	−9.97E-02
Hapmap27083-BTC-041166	38,212,942	*LCORL*	2.58E-07	1.52E-03	0.36	7.50E-02
Hapmap23507-BTC-041133	38,233,089	*LCORL*	3.89E-07	2.01E-03	0.36	7.39E-02
Hapmap31285-BTC-041097	38,256,890	*LCORL*	4.11E-11	4.24E-07	0.48	−9.30E-02
Hapmap33628-BTC-041023	38,326,148	*LCORL*	7.12E-12	9.80E-08	0.46	−9.85E-02
Hapmap25414-BTC-034877	38479644	NA	2.45E-05	7.78E-02	0.27	6.71E-02
Hapmap32207-BTC-034871	38,500,210	NA	2.45E-05	7.78E-02	0.27	6.71E-02
Hapmap28546-BTC-072715	38,558,527	NA	4.84E-07	2.22E-03	0.38	7.38E-02
Hapmap27537-BTC-060891	38,638,963	NA	1.04E-05	3.90E-02	0.39	6.68E-02
Hapmap31044-BTC-071337	38,729,867	NA	2.38E-09	1.94E-05	0.46	8.66E-02

SNP name, position, the gene in which they are located (if any), significance levels both unadjusted (P*) and corrected for multiple testing (Q*), allelic frequencies (fB) and effects of the minor allele (βB).

### Association of Additional SNPs Located within LAP3, NCAPG and LCORL Genes

**Figure 3:**
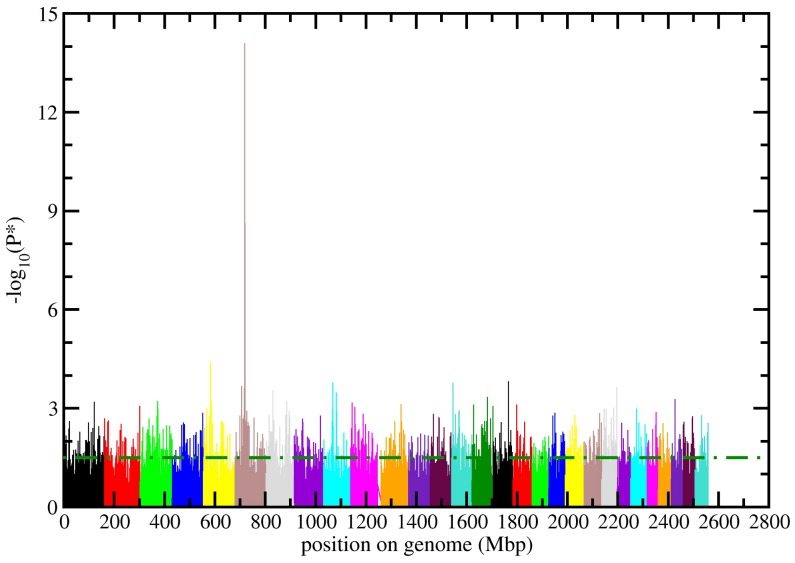
Descriptive histograms for the reduced population of 247 sires used for additional genotyping of SNPs within the *LAP3*, *NCAPG* and *LCORL* genes after the GWAS. Left Panel: histograms of EBVs for direct calving ease distributed in five equally spaced classes. Right panel: histogram of kinship coefficients between sires in five equally spaced classes. In both panels, the number of sires belonging to each class is plotted as a function of classes.

Based on the results of genome wide scan we decided to genotype additional SNPs in the genes located in the region where we found a strong association of the Illumina Bovine SNP50 with direct calving ease. We therefore selected, from NCBI dbSNP (http://www.ncbi.nlm.nih.gov/snp/) and from the literature [12], [13], 12 SNPs in *LAP3* (only one marker from the Illumina BovineSNP50 panel was located in this gene), 5 in *NCAPG*, and 5 in *LCORL* (see Table S1 for complete information on these additional SNPs). Markers were genotyped on the Piedmontese population, leaving out sires discarded in the GWAS step. However, due to limited availability of biological samples, we were able to obtain further DNA only from 247 subjects of the original population. Data editing of these 22 additional SNPs yielded the following results: of the 247 starting sires, 6 were discarded for having a call rate <95%; 4 markers were excluded because the call rate was <95% and 10 markers were excluded for being monomorphic or for having Minimum Allele Frequency (MAF) below 5%. Checking the EBV distribution of this partially reduced population we verified that it was closely matching the whole one used for GWAS and thus no significant bias should have been introduced by the missing subjects. Figure 3 shows histograms, analogous to those in Figure 1, with the distribution of trait values and kinship coefficients for these 247 sires; in particular, it can be seen that the greater part of pairs of sires shows a slightly negative kinship (Figure 3, right panel) as seen in the whole population (Figure 1, right panel). The results of the statistical analysis are shown in Table 2. All SNPs resulted associated to calving ease with significance levels ranging from 8.34E-08 to 7.42E-04. The strongest association was observed for the SNP *rs110251642* located in the *NCAPG* gene but overall the most important signal was observed for the *LAP3* gene since more than half of the markers within its sequence resulted associated with the studied trait. Moreover, with the exception of *rs4125598* (P* = 6.955E-07, β = 7.335E-02), all SNPs shared the same P* and β implying that these markers are in high LD. The average LD estimation among *LAP3* and *LCORL* SNPs was r^2^ = 0.653 (higher than that obtained analyzing SNPs located within the same genes using the BovineSNP50 v1 panel) but this value dropped to r^2^ = 0.383 if all significant SNPs located in the peak on BTA6 (i.e. from both GWAS and additional genotyping data) were included. It is also worth to observe that SNPs located in *LAP3* gene showed the highest LD (r^2^ = 0.783) in agreement with the GWAS results. The significant SNP in the region explained about 22.8% of the EBV variance (Principal Components Regression was used to cope with correlation among SNPs).

**Table 2:** SNP name, gene name, region within the gene, significance level (P*) and minor allele effect (βB) for the SNPs selected in the *LAP3*, *NCAPG* and *LCORL* genes and significantly associated to direct calving ease.

SNP	gene	gene region	P*	βB
*rs41255598*	*LAP3*	non synonymous codon	7.419E-04	1.658E-02
*rs43702364*	*LAP3*	intron 12 (24428-24878)	6.955E-07	7.335E-02
*rs43702363*	*LAP3*	intron 12	6.955E-07	7.335E-02
*rs43702362*	*LAP3*	intron 12	6.955E-07	7.335E-02
rs110839532	*LAP3*	3'UTR	6.955E-07	7.335E-02
rs109241256	*LAP3*	3'UTR	6.955E-07	7.335E-02
rs41255599	*LAP3*	3'UTR	6.955E-07	7.335E-02
*r*s110251642	*NCAPG*	non synonymous codon exon 17	8.341E-08	-8.252E-02
*rs110422856*	*NCAPG*	intron variant, splice region variant	4.871E-04	5.394E-02
*rs109572301*	*LCORL*	3'UTR variant	4.692E-04	5.414E-02

Where available, rs number links to NCBI dbSNP page for the relative SNP.

## Discussion

Today, selection objectives in agriculture pay a great deal of attention to robustness, longevity, welfare and, more generally, sustainability of production. Calving ease is a functional trait having a large impact on animal health and farm profitability, and it also make feasible to breeders the exploitation of sustainable livestock systems [14]. However, antagonistic relationships between calving performance and beef production traits [15] make the classical breeding schemes not efficient and highlights the need for improved selection strategies. The possibility to select individuals based on alleles at genes affecting calving performances may help to overcome this problem. Moreover, Piedmontese is a breed carrying the G1056A mutation in the *myostatin* gene [11], [16], [17], which affects calving ease, although less severely than in other breeds carrying disruptive mutations, as Belgian Blue [16] and Marchigiana [18]. Furthermore, estimates of heritability of maternal and direct calving related traits for the Italian Piedmontese population are, like many other estimates in several other breeds, between 0.19 and 0.08 depending of the number of parities and the type of effect considered [19] and this proves the difficulty of establishing selection strategies based on traditional breeding programs. Therefore, several attempts have been made to use DNA information for improving this trait. In cattle, QTLs worth of further study, affecting calving ease and foetus growth-related traits, are described on various chromosomes. A total of 61 and 38 QTLs for direct and maternal calving ease, respectively, are reported in public databases (http://www.animalgenome.org/cgi-bin/QTLdb/BT/index). Pausch and collaborators identified by genome-wide association two major loci on BTA 14 and 21 affecting calving ease and growth-related traits, respectively [10]. Other studies point to the existence of QTLs for calving ease and foetal growth on BTA 6 in various cattle populations [20], [21], [22].

In this study, we performed a genome wide scan of Piedmontese cattle using a medium density chip featuring about 54K SNPs. The chip analysis clearly showed a cluster of 13 SNPs in a 0.9 Mb wide region of BTA6 significantly associated (P*<0.001) with direct calving ease. Three interesting genes are located in this region: *LAP3*, encoding for a leucine aminopeptidase, which is responsible of the oxytocin hydrolysis [12], [13]; *NCAPG*, encoding for a non-SMC condensin I complex, which has been associated in cattle with foetal growth and carcass size [23], [24]; *LCORL*, encoding for a ligand dependent nuclear receptor co-repressor-like, which affects height in humans and also controls stature in cattle in high correlation with *NCAPG* [25]. In a crossbred population of beef cattle, Snelling and collaborators identified a region on BTA6 (37.96–38.53 Mb, on the Btau_4.0) associated with feed intake, gain, meat and carcass traits [26], [27], [28], [29]. Four markers typed in *LCORL* were also found associated with carcass weight in Japanese Black steers [24]. We considered *LAP3*, *NCAPG* and *LCORL* as potential positional and functional candidate genes on BTA6 for direct calving ease. Thus, we chose to enrich the SNP set in these genes to be genotyped on the population. Two out of five SNPs in *NCAPG* show a weak association to direct calving ease and a positive effect linked to the homozygote genotypes for the major allele (T/T in *rs109570900* and C/C in *rs110251642*). Recently it has been shown that a polymorphism in the *NCAPG* gene (*rs109570900,* 1326T>G) is significantly associated to an increase in bovine carcass weight at puberty [24], [28], [33]. Only one of the 5 markers selected in *LCORL* (*rs109572301*) was significantly associated with direct calving ease. *LCORL* is immediately adjacent to the *NCAPG* gene; the *LCORL/NCAPG* locus has been associated with human height [25], [30], [31], [32], with feed intake, gain, meat and carcass traits in beef cattle [28], and very recently with size variation in horses [34], [35]. Among the three genes included in the statistically associated region, *LAP3* is the one showing the most suggestive involvement with the trait. It results significant considering both SNPs in the Illumina BovineSNP50 chip and the further genotyped SNPs. Moreover, the SNPs within its sequence show the highest Linkage Disequilibrium, which is probably due to an increased fitness or to breeders selection. It is worth to observe that these results add relevant information to those obtained in the GWAS since the Bovine SNP50 v1 panel contains only one marker in *LAP3* and the strongest signal was obtained for the *LCORL* gene. However, it is not possible to draw definitive conclusions about the relative importance of *LCORL* or *NCAPG* as compared with *LAP3* since most markers genotyped within these genes were not usable as being monomorphic or with MAF<0.05. We cannot rule out that the region was subject to selective sweep that dropped out one allele. *LAP3* is involved in oxytocin metabolism and hence in maternal calving effects, nevertheless we did not find an association with this trait (data not shown). This lack of association is probably due to the scarcity of data since EBV records for maternal calving effects were available for less than one hundred sires and with lower accuracy since calves outnumber dams.

Finally, our genome-wide association study shows that EBVs of traits with low heritability are still able to capture the molecular features of the genome. These can be exploited by a Gene Assisted Selection (GAS), that is comparably much faster and more efficient than the selection based on EBV. Furthermore, traits with low heritability should not be disregarded because their relative objectives of selection can only be reached in a long time, indeed they may be successfully dissected to reveal important insights at molecular level.

## Materials and Methods

### Animals and EBV Data

Genomic DNA was extracted from semen of 323 Piedmontese bulls using the NucleoSpin Tissue kit (Macherey & Nagel) according to manufacturer’s instruction. DNA was checked for quality on agarose gel and quantified using a DTX microplate reader (Beckman Coulter) after staining with Picogreen (Invitrogen). This Italian breed has been selected for beef production and almost all individuals carry muscular hyperplasia [11]. Estimated Breeding Values (EBVs) were provided by the Italian Piedmontese National Breeders Association (ANABORAPI, http://www.anaborapi.it/). The sample includes almost all Piedmontese bulls for which data were available and thus it represents the most complete population available for the association analysis for this breed.

### SNP Chip and Genotyping

In total, 323 individuals were genotyped using BovineSNP50 BeadChips (Illumina, San Diego, CA, USA). The Illumina BovineSNP50 array contains 54,001 SNPs distributed across the entire genome, with an average SNP spacing of 51 Kb and a proportion of known chromosome positions of about 97%. Genotyping was outsourced to Geneseek, USA (www.geneseek.com). SNP calls accurate to more than 99.8% were obtained on average. The SNP positions within each chromosome were based on the *Bos taurus* genome assembly Btau_4.0 [36].

### Data Editing and Genome-wide Analysis

Sires and markers with a call rate under 95% were discarded, as well as SNPs having a minor allele frequency (MAF) <2.5%. Sires were checked for abnormally high autosomal heterozygosity (FDR 1%) [37], [38]. Hardy Weinberg Equilibrium was checked on the filtered data set [39]; markers were removed from the data set if the P-value was <0.01. Kinship among sires was estimated directly from genomic data as proposed by Astle and Balding [40]. Association of SNPs with phenotypes was performed using the Family based Test for Association to reduce computing time [41]. Kinship among sires was treated as a random effect, while SNP genotypes were considered as fixed effects. The model used was:
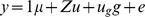
where *μ* is the mean and *1* is a vector of ones, *Zu* accounts for polygenic effects, *u_g_* is the effect of the marker under investigation and *g* denotes a genotype, *e* is a vector of residuals. Polygenic effects were estimated by calculating genomic kinship between sires using the complete set of filtered SNPs [42], [43]. Genomic control was applied and the inflation factor λ was calculated and used to correct significance levels [44]. False Discovery Rate control was performed using the Benjamini-Hochberg method [45] using a FDR of 0.1. LD in selected regions was estimated calculating the r^2^ coefficient between SNPs using the approach proposed by Hao [46].

### Genotyping of Additional SNPs on BTA6

Since the genome wide analysis pointed to a ∼0.9 Mb region associated to direct calving ease (see Results) we searched for further SNPs not represented in the Illumina BovineSNP50 panel in genes included in this region to validate and enrich the results of the genome wide scan. We selected 12 SNPs in the *LAP3* gene, 5 in the *NCAPG* gene and 5 in *LCORL* (Table S1). For *LAP3* three markers were described by Zheng [12], while nine SNPs were selected by screening *in silico* NCBI dbSNP (http://www.ncbi.nlm.nih.gov/snp/). Three SNPs in *NCAPG* gene were described by Setoguchi [13], [24], other two were selected in NCBI dbSNP. The five markers in *LCORL* gene were all selected in NCBI dbSNP. The 22 SNPs were genotyped on 247 bulls from the whole population from which we could recover biological samples. SNP genotyping was outsourced to Kbiosciences (www.kbiosciences.co.uk). Data were filtered using the same criteria illustrated above for the SNP chip data. Association between these SNPs and EBVs for direct calving ease was performed using the same statistical model employed for the genome-wide scan but without correcting for inflation factor or for multiple testing since in this case SNPs were limited; however the full kinship matrix obtained with data from the BovineSNP50 panel and the bulls for which additional genotyping was available was used in the calculations.

The variance explained by the SNPs was evaluated using a Principal Component Analysis regression to cope with correlation (Linkage Disequilibrium) among markers [47].

## Supporting Information

Figure S1 QQ-plot of significance levels for the GWAS scan prior to normalization.
(PNG)

Table S1 **Additional SNPs genotyped in the** ***LAP3*** **,** ***NCAPG*** **and** ***LCORL*** **genes.** The *rs* number, the gene region and the reference are shown.
(PDF)

## References

[1] Meijering A (1984) Dystocia and stillbirth in cattle A review of causes, relations and implications. Livest Prod Sci 11: 143–177.

[2] Freer B (2008) Easy calving: not so difficult. Hereford Breed J 176–177.

[3] Van Tassell CP, Wiggans GR, Misztal I (2003) Implementation of a sire-maternal grandsire model for evaluation of calving ease in the United States. J Dairy Sci 86: 3366–3373. 14594257 10.3168/jds.S0022-0302(03)73940-X

[4] Holmberg M, Andersson-Eklund L (2006) Quantitative trait loci affecting fertility and calving traits in Swedish dairy cattle. J Dairy Sci 89: 3664–3671. 16899702 10.3168/jds.S0022-0302(06)72406-7

[5] Druet T, Fritz S, Boussaha M, Ben-Jemaa S, Guillaume F, et al (2008) Fine mapping of quantitative trait loci affecting female fertility in dairy cattle on BTA03 using a dense single-nucleotide polymorphism map. Genetics 178: 2227–35. 18430945 10.1534/genetics.107.085035 PMC2323811

[6] Thomasen JR, Guldbrandtsen B, Sørensen P, Thomsen B, Lund MS (2008) Quantitative trait loci affecting calving traits in Danish Holstein cattle. J Dairy Sci 91: 2098–105. 18420641 10.3168/jds.2007-0602

[7] Höglund JK, Guldbrandtsen B, Su G, Thomsen B, Lund MS (2009) Genome scan detects quantitative trait loci affecting female fertility traits in Danish and Swedish Holstein cattle. J Dairy Sci 92: 2136–43. 19389971 10.3168/jds.2008-1104

[8] Brand B, Baes C, Mayer M, Reinsch N, Seidenspinner T, et al (2010) Quantitative trait loci mapping of calving and conformation traits on *Bos taurus* autosome 18 in the German Holstein population. J Dairy Sci 93: 1205–15. 20172241 10.3168/jds.2009-2553

[9] Sahana G, Guldbrandtsen B, Lund MS (2011) Genome-wide association study for calving traits in Danish and Swedish Holstein cattle. J Dairy Sci 94: 479–86. 21183059 10.3168/jds.2010-3381

[10] Pausch H, Flisikowski K, Jung S, Emmerling R, Edel C, et al (2011) Wide Association Study Identifies Two Major Loci Affecting Calving Ease and Growth-Related Traits in Cattle. Genetics 187: 289–297. 21059885 10.1534/genetics.110.124057 PMC3018322

[11] Wiener P, Smith JA, Lewis AM, Woolliams JA, Williams JL (2002) Muscle-related traits in cattle: The role of the myostatin gene in the South Devon breed. Genet. Sel. Evol. 2: 221–232. 10.1186/1297-9686-34-2-221 PMC2705429 12081809

[12] Zheng X, Ju Z, Wang J, Li Q, Huang J, et al (2010) Single nucleotide polymorphisms, haplotypes and combined genotypes of LAP3 gene in bovine and their association with milk production traits. Molecular Biology Reports 38: 4053–4061. 21110109 10.1007/s11033-010-0524-1

[13] Eberlein A, Takasuga A, Setoguchi K, Pfuhl R, Flisikowski K, et al (2009) Dissection of genetic factors modulating fetal growth in cattle indicates a substantial role of the non-SMC condensin I complex, subunit G (NCAPG) gene. Genetics 183: 951–964. 19720859 10.1534/genetics.109.106476 PMC2778990

[14] Thompson PB, Nardone A (1999) Sustainable livestock production: methodological and ethical challenges. Livestock Production Science 61: 111–119.

[15] Albera A, Groen AF, Carnier P (2004) Genetic relationships between calving performance and beef production traits in Piemontese cattle. J Anim Sci 82: 3440–3446. 15537762 10.2527/2004.82123440x

[16] Mc Pherron AC, Lee SL (1997) Double muscling in cattle due to mutations in the myostatin gene. Proc Natl Acad Sci U S A 94: 12457–12461. 9356471 10.1073/pnas.94.23.12457 PMC24998

[17] Grobet L, Poncelet D, Royo LJ, Brouwers B, Pirottin D, et al (1998) Molecular definition of an allelic series of mutations disrupting the myostatin function and causing double-muscling in cattle. Mammalian Genome 9: 210–213. 9501304 10.1007/s003359900727

[18] Marchitelli C, Savarese MC, Crisà A, Nardone A, Marsan PA, et al (2003) Double muscling in Marchigiana beef breed is caused by a stop codon in the third exon of myostatin gene. Mamm Genome. 6: 392–395. 10.1007/s00335-002-2176-5 12879361

[19] Carnier P, Albera A, Dal Zotto R, Groen AF, Bona M, et al (2000) Genetic parameters for direct and maternal calving ability over parities in Piedmontese cattle. J Animal Science 78: 2532–2539. 10.2527/2000.78102532x 11048917

[20] Schrooten C, Bink MC, Bovenhuis H (2004) Whole genome scan to detect chromosomal regions affecting multiple traits in dairy cattle. J Dairy Sci 87: 3550–60. 15377635 10.3168/jds.S0022-0302(04)73492-X

[21] Kneeland J, Li C, Basarab J, Snelling WM, Benkel B, et al (2004) Identification and fine mapping of quantitative trait loci for growth traits on bovine chromosomes 2, 6, 14, 19, 21, and 23 within one commercial line of *Bos taurus* . J Anim Sci 82: 3405–14. 15537758 10.2527/2004.82123405x

[22] Gutiérrez-Gil B, Williams JL, Homer D, Burton D, Haley CS, et al (2009) Search for quantitative trait loci affecting growth and carcass traits in a cross population of beef and dairy cattle. J Anim Sci 87: 24–36. 18791160 10.2527/jas.2008-0922

[23] Olsen HG, Lien S, Gautier M, Nilsen H, Roseth A, et al (2005) Mapping of a milk production quantitative trait locus to a 420-kb region on bovine chromosome 6. Genetics 169: 275–283. 15466433 10.1534/genetics.104.031559 PMC1448861

[24] Setoguchi K, Furuta M, Hirano T, Nagao T, Watanabe T, et al (2009) Cross-breed comparisons identified a critical 591-kb region for bovine carcass weight QTL (CW-2) on chromosome 6 and the Ile-442-Met substitution in NCAPG as a positional candidate. BMC Genetics 10: 43. 19653884 10.1186/1471-2156-10-43 PMC2736976

[25] Pryce JE, Hayes BJ, Bolormaa S, Goddard ME (2011) Polymorphic regions affecting human height also control stature in cattle. Genetics 187: 981–984. 21212230 10.1534/genetics.110.123943 PMC3048786

[26] Snelling WM, Allan MF, Keele JW, Kuehn LA, McDanel T, et al (2010) Genome-wide association study of growth in crossbred beef cattle. Journal of Animal Science 88: 837–848. 19966163 10.2527/jas.2009-2257

[27] Snelling WM, Allan MF, Keele JW, Kuehn LA, Thallman RM, et al (2011) Partial-genome evaluation of postweaning feed intake and efficiency of crossbred beef cattle. Journal of Animal Science 89: 1731–1741. 21297062 10.2527/jas.2010-3526

[28] Lindholm-Perry AK, Sexten AK, Kuehn LA, Smith TPL, King DA, et al (2011) Association, effects and validation of polymorphisms within the NCAPG - LCORL locus located on BTA6 with feed intake, gain, meat and carcass traits in beef cattle. BMC Genetics 12: 103. 22168586 10.1186/1471-2156-12-103 PMC3287254

[29] Lindholm-Perry AK, Kuehn LA, Snelling WM, Smith TPL, Ferrell CL, et al.. (2012) Genetic markers on BTA14 predictive for residual feed intake in beef steers and their effects on carcass and meat quality traits. Animal Genetics. Article in Press. 10.1111/j.1365-2052.2011.02307.x 22497335

[30] Paternoster L, Howe LD, Tilling K, Weedon MN, Freathy RM, et al (2011) Adult height variants affect birth length and growth rate in children. Hum Mol Genet. 20: 4069–4075. 10.1093/hmg/ddr309 PMC3177650 21757498

[31] Weedon MN, Lango H, Lindgren CM, Wallace C, Evans DM, et al (2008) Genome-wide association analysis identifies 20 loci that influence adult height. Nature Genetics 40: 575–583. 18391952 10.1038/ng.121 PMC2681221

[32] Gudbjartsson DF, Walters GB, Thorleifsson G, Stefansson H, Halldorsson BV, et al (2008) Many sequence variants affecting diversity of adult human height. Nature Genetics 40: 609–615. 18391951 10.1038/ng.122

[33] Setoguchi K, Watanabe T, Weikard R, Albrecht E, Kühn C, et al (2011) The SNP c1326T>G in the non-SMC *condensin I complex, subunit G* (NCAPG) gene encoding a pIle442Met variant is associated with an increase in body frame size at puberty in cattle. Animal Genetics 42(6): 650–655. 22035007 10.1111/j.1365-2052.2011.02196.x

[34] Makvandi-Nejad S, Hoffman GE, Allen JJ, Chu E, Gu E, et al.. (2012) Four Loci Explain 83% of Size Variation in the Horse. PLoS One. 7(7): e39929. Published online 2012 July. 10.1371/journal.pone.0039929 PMC3394777 22808074

[35] Signer-Hasler H, Flury C, Haase B, Burger D, Simianer H (2012) Genome-Wide Association Study Reveals Loci Influencing Height and Other Conformation Traits in Horses. PLoS One 7(5): e37282. Published online 2012 May 16. 10.1371/journal.pone.0037282 PMC3353922 22615965

[36] Liu Y, Qin X, Song X-ZH, Jiang H, Shen Y, et al. (2009) *Bos taurus* genome assembly. BMC Genomics 10, art no 180. 10.1186/1471-2164-10-180 PMC2686734 19393050

[37] Balding DJ (2006) A tutorial on statistics methods for population association studies. Nat Rev Gen 7: 781–791. 10.1038/nrg1916 16983374

[38] Pearson TA, Manolio TA (2008) How to interpret a Genome-wide Association study. JAMA 299(11): 1335–1344. 18349094 10.1001/jama.299.11.1335

[39] Wigginton JE, Cutler DJ, Abecasis GR (2005) A Note on Exact Tests of Hardy-Weinberg Equilibrium. Am J Hum Genet 76: 887–883. 15789306 10.1086/429864 PMC1199378

[40] Astle W, Balding DJ (2009) Population structure and cryptic relatedness in genetic association studies. Statistical Science 24: 451–471.

[41] Chen WM, Abecasis GR (2007) Family-based association tests for genomewide association scans. Am J Hum Genet 81: 913–26. 17924335 10.1086/521580 PMC2265659

[42] Aulchenko YS, de Koning DJ, Haley C (2007) Genomewide rapid association using mixed model and regression: a fast and simple method for genome-wide pedigree-based quantitative trait loci association analysis. Genetics 177: 577–85. 17660554 10.1534/genetics.107.075614 PMC2013682

[43] Amin N, van Duijn CM, Aulchenko YS (2007) A genomic background based method for association analysis in related individuals PLoS ONE. 12: 1274. 10.1371/journal.pone.0001274 PMC2093991 18060068

[44] Devlin B, Roeder K (1999) Genomic Control for Association Studies. Biometrics 55: 997–1004. 11315092 10.1111/j.0006-341x.1999.00997.x

[45] Benjamini Y, Hochberg Y (1995) Controlling the false-discovery rate: a practical and powerful approach to multiple testing. J Roy Statist Soc Ser B 57: 289–300.

[46] Hao K, Di X, Cawley S (2007) LdCompare: rapid computation of single- and multiple-marker r2 and genetic coverage. Bioinformatics 23: 252–254. 17148510 10.1093/bioinformatics/btl574

[47] Mevik B-H, Wehrens R (2007) The pls Package: Principal Component and Partial Least Squares Regression in R, Journal of Statistical Software, 18, issue i02.

