# Supplementary figures and images for: Identification of a Short Region on Chromosome 6 Affecting Direct Calving Ease in Piedmontese Cattle Breed
Source: PLoS One. 2012 Dec 4;7(12):e50137. doi: 10.1371/journal.pone.0050137 (PMC3514265; doi:10.1371/journal.pone.0050137)

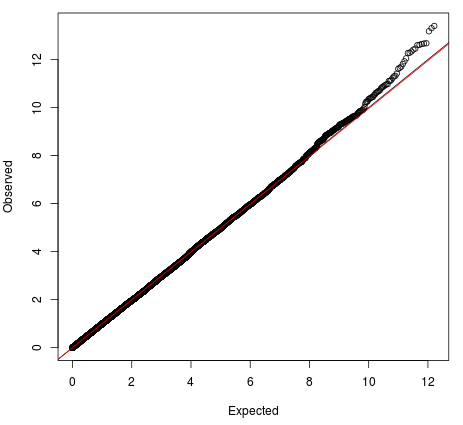

Supplement: Figure S1 — QQ-plot of significance levels for the GWAS scan prior to normalization. (PNG) [file pone.0050137.s001.png]
