# Supplementary material for: Identification of a Short Region on Chromosome 6 Affecting Direct Calving Ease in Piedmontese Cattle Breed
Source: PLoS One. 2012 Dec 4;7(12):e50137. doi: 10.1371/journal.pone.0050137 (PMC3514265; doi:10.1371/journal.pone.0050137)
Supplement: Table S1 — Additional SNPs genotyped in the LAP3 , NCAPG and LCORL genes. The rs number, the gene region and the reference are shown. (PDF) [file pone.0050137.s002.pdf]

Identification of a short region in chromosome 6 affecting direct calving ease in Piedmontese cattle breed – Supplementary material

Silvia Bongiorni[1,§], Giordano Mancini[2,§,*], Giovanni Chillemi[2], Lorraine Pariset[1] and Alessio Valentini[1]

Table S1

Additional SNPs genotyped in the *LAP3*, *NCAPG* and *LCORL* genes. The *rs* number, the gene region and the reference are shown.

SNPs in *LAP3*	Gene region	Reference
rs110839532	3'UTR	NCBI dbSNP
rs43702361	3'UTR	NCBI dbSNP
rs109241256	3'UTR	NCBI dbSNP
rs41255599	3'UTR	NCBI dbSNP
rs41255598	non synonymous codon	NCBI dbSNP
rs43702364	intron 12 (24428-24878)	Zheng et al., 2010
rs43702363	intron 12	Zheng et al., 2010
rs43702362	intron 12	Zheng et al., 2010
rs137589964	frameshift variant	NCBI dbSNP
rs133320800	non synonymous codon, splice region variant	NCBI dbSNP
rs109133649	non synonymous codon	NCBI dbSNP
rs137449965	intron variant, splice region variant	NCBI dbSNP
**SNPs in *NCAPG***	**Gene region**	**Reference**
rs109570900	non synonymous codon exon 9	Setoguchi et al. 2009
rs110251642	non synonymous codon exon 17	Setoguchi et al. 2009
rs110757748	intron	Setoguchi et al. 2009
rs133801456	non synonymous codon	NCBI dbSNP
rs110422856	intron variant, splice region variant	NCBI dbSNP
**SNPs in *LCORL***	**Gene region**	**Reference**
rs109572301	3’UTR variant	NCBI dbSNP
rs110513685	non synonymous codon	NCBI dbSNP
rs108961108	stop gained	NCBI dbSNP
rs110125137	5’UTR variant	NCBI dbSNP
rs133803445	5’UTR variant	NCBI dbSNP
